# Analysis of different levels of positive end-expiratory pressure during lung retrieval for transplantation: an experimental study

**DOI:** 10.1590/1414-431X20198585

**Published:** 2019-07-15

**Authors:** W.A. Gonçalves-Ferri, A. Jauregui, F.P. Martins-Celini, I. Sansano, A.T. Fabro, E.M.F. Sacramento, D.C. Aragon, J.M. Ochoa

**Affiliations:** 1 Departamento de Pediatria, Faculdade de Medicina de Ribeirão Preto, Universidade de São Paulo, Ribeirão Preto, SP, Brasil; 2 Department of Thoracic Surgery, Hospital Vall d'Hebron, Barcelona, Spain; 3 Department of Pathology, Hospital Vall d'Hebron, Barcelona, Spain; 4 Departamento de Patologia, Faculdade de Medicina de Ribeirão Preto, Universidade de São Paulo, Ribeirão Preto, SP, Brasil

**Keywords:** Anesthesia, Lung transplantation, Mechanical ventilation, Pulmonary recruitment, PEEP

## Abstract

Atelectasis and inadequate oxygenation in lung donors is a common problem during the retrieval of these organs. Nevertheless, the use of high positive end-expiratory pressure (PEEP) is not habitual during procedures of lung retrieval. Twenty-one Sprague-Dawley male consanguineous rats were used in the study. The animals were divided into 3 groups according to the level of PEEP used: low (2 cmH_2_O), moderate (5 cmH_2_O), and high (10 cmH_2_O). Animals were ventilated with a tidal volume of 6 mL/kg. Before lung removal, the lungs were inspected for the presence of atelectasis. When atelectasis was detected, alveolar recruitment maneuvers were performed. Blood gasometric analysis was performed immediately. Finally, the lungs were retrieved, weighed, and submitted to histological analysis. The animals submitted to higher PEEP showed higher levels of oxygenation with the same tidal volumes PO_2_=262.14 (PEEP 2), 382.4 (PEEP 5), and 477.0 (PEEP 10). The occurrence of atelectasis was rare in animals with a PEEP of 10 cmH_2_O, which therefore required less frequent recruitment maneuvers (need for recruitment: PEEP 2=100%, PEEP 5 =100%, and PEEP 10=14.3%). There was no change in hemodynamic stability, occurrence of pulmonary edema, or other histological injuries with the use of high PEEP. The use of high PEEP (10 cmH_2_O) was feasible and probably a beneficial strategy for the prevention of atelectasis and the optimization of oxygenation during lung retrieval. Clinical studies should be performed to confirm this hypothesis.

## Introduction

Knowledge about lung transplantation has greatly progressed over the last decades. However, mean survival after transplantation is 6 years according to data of the International Society for Heart and Lung Transplantation (ISHLT) (1). Despite the advances obtained, post-transplant pulmonary complications such as primary graft dysfunction still occurs in 25% of cases, impairing the results (2).

Several techniques for lung preservation and minimization of undesirable changes in the organs to be transplanted have been investigated. A method of relatively low cost is the use of ventilatory strategies in order to preserve pulmonary tissues. Among the lung-protective strategies that may minimize lung injuries are the use of low tidal volume, alveolar recruitment, and adequate positive end- expiratory pressure (PEEP) levels. However, these strategies have not been well established (3–5).

The use of adequate PEEP is a lung-protective ventilation technique that is being shown to be effective and has been suggested in some protocols of ventilation for brain-dead donors (6). PEEP can prevent airway collapse and improve residual functional capacity, thus reducing the proportion of non-aerated alveoli at the end of expiration. However, very high PEEP levels may cause alveolar hyperdistention with worsening of ventilation-perfusion balance and hemodynamic repercussions (7,8).

The main benefit of adequate PEEP in lung donors would be the prevention of formation of atelectasis, a quite frequent complication during lung retrieval and perfusion. Atelectasis of lungs to be transplanted can favor rapid perfusion due to the reduced vascular area and lead to high perfusion pressure in ischemic lungs, with consequent pulmonary injury and edema. Thus, improving alveolar recruitment before lung removal appears to be a safe procedure that may help prevent injuries due to reperfusion (9).

Several methods for the treatment of atelectasis have been studied, with most investigations using acute alveolar recruitment maneuvers (3,9,10 [Bibr B11]). Alveolar recruitment maneuvers are also the procedures more frequently used in clinical practice to treat atelectasis during lung retrieval, yielding many benefits such as improved pulmonary compliance and a reduced need for ventilatory parameters. However, they may provoke the release of inflammatory mediators causing pulmonary injury (9–12).

Therefore, the use of a ventilatory strategy that causes the smallest possible number of atelectasis areas and avoids the need for their treatment with recruitment maneuvers would be ideal. The use of high PEEP throughout the lung retrieval process could be an option. Therefore, our hypothesis was that higher PEEP would lead to better pulmonary recruitment and consequently better oxygenation and less need for invasive ventilatory maneuvers, contributing to the quality of the organ to be transplanted.

The objective of the present study was to evaluate the use of different levels of PEEP throughout the pulmonary retrieval process in an animal model.

## Material and Methods

### Animals

Twenty-one Sprague-Dawley male consanguineous rats weighing 320–450 g were used as lung donors and housed under optimal conditions. All animals were treated in accordance with the “Guidelines for the use and care of laboratory animals” published by the Spanish National Health Institute. The study was approved by the Ethics Committee for Animal Experimentation and Well-being of Vall d’Hebron University Hospital (IAACUC 22/2012).

### Lung retrieval technique

Right lungs from donor rats were used in the study. Animals were randomized into 3 groups by the drawing of sealed opaque envelopes, according to the PEEP level: Group A, ventilated with a PEEP of 2 cmH_2_O (low); Group B, ventilated with a PEEP of 5 cmH_2_O (moderate), and Group C, ventilated with a PEEP of 10 cmH_2_O (high).

Animals were anesthetized with 0.25 mg/kg subcutaneous medetomidine (Domtor^®^, Pfizer, Spain) and 50 mg/kg intramuscular ketamine (Ketolar 500^®^, Pfizer). Animals were intubated with an Abbocath 14G catheter (BD, USA) and placed in supine position. Immediately after intubation, mechanical ventilation was initiated.

### Ventilatory strategies and monitoring

Ventilation of the lung was initiated with a tidal volume of 6–8 mL/kg, a driving pressure of 12–14 cmH_2_O, inspiratory-expiratory index ratio of 1:2, and a frequency of 60 breaths per minute with a Servo ventilator 300 (Siemens, Germany) for 10 min (time for lung removal). Gas temperature was maintained at 36°C.

The leak was determined through the difference between expiratory and inspiratory tidal volumes (VTe – VTi). Only animals with a tracheal leak of less than 10% of inspiratory tidal volume were ventilated. The temperature and heart rate of the animals was monitored with a multiparametric monitor for rodents (PhysioSuite^®^ for Mice & Rats, Kent Scientific, USA).

For gasometry, catheterization and extraction of 0.05 mL of blood from the left ventricle for basal gas analysis with I-Stat^®^ (Abbot Laboratories, USA) were performed. For organ removal, the thymus was excised and the ascending aorta was dissected and clamped with a 4-mm microclamp (Yasargil^®^, Medicom, Germany). After exsanguination and cardiac arrest, anterograde perfusion (preservation solution) was performed with an incision in the base of the cone of the pulmonary artery using an Abbocath 16G catheter with 20 mL of cold preservation solution of low-potassium dextran glucose (Perfadex^®^, XVIVO Perfusion, Sweden) at a height of 30 cm.

Before removal, the lung was inspected by two investigators; if there were areas of pulmonary collapse, characterized by color alteration, associated with loss of lung volume, with parenchyma retraction, it was considered as presence of atelectasis and the recruitment maneuver was performed with an additional increase of 5 cmH_2_O in PEEP and in peak pressure for 5 min. The PEEP used in the recruitment maneuver was 7 for group PEEP 2, 10 for group PEEP 5, and 15 for group PEEP 10. The driving pressure was maintained at 14 cmH_2_O during recruitment. Recruitment maneuvers followed the principle of recruitment monographs described by Meade et al. (13). The FiO_2_ used was 100%. The researchers were not blinded to the value of PEEP.

The cardiopulmonary block was extracted and separated from the esophagus. After dissection, the right lung was weighed on a high precision scale, submerged in 10% formaldehyde, and sent for histological examination.

### Histological analysis

Formalin-fixed lungs were sectioned and embedded in paraffin. One slide of each was stained with hematoxylin-eosin and observed at an optical microscope by a pathologist (blinded about the groups). Histological deviations from normality were recorded and graded 0–4, when possible.

Results of the lung interventions was done according to the guidelines of the Acute Lung Injury in Animals Study Group (14).

### Statistical analysis

ANOVA was used to compare the mean data of the groups. When the null hypothesis was rejected for each variable, orthogonal contrasts were estimated for multiple comparisons. The Fisher’s exact test was applied to determine the association between groups and the need for recruitment.

Poisson regression models were adjusted for the counting variables such as pneumonitis, cellular bronchiolitis, and air space collapse, allowing multiple comparisons of the means between groups. The software used was SAS 9.3 (SAS Institute, USA). The level of significance was set at 5%.

## Results

Twenty-one animals with a mean weight of 383 g were used. Groups A, B, and C had respective mean weights of 361, 400, and 391 g.

The group with PEEP of 10 presented better oxygenation (PO_2_) than the group with PEEP of 2. There was no difference in oxygenation between PEEP 10 and PEEP 5, and PEEP 10 induced hypercapnia and respiratory acidosis (Table 1).

**Table 1:** Comparison of gasometry mean values for different final positive end-expiratory pressure (PEEP) groups.

Gasometry	PEEP 2	PEEP 5	PEEP 10
pH	7.2 (0.09)*	7.2 (0.01)	7.1 (0.09)
PCO_2_ (mmHg)	72.0 (19.5)*	73.0 (14.6)^#^	100.0 (17.3)
PO_2_ (mmHg)	262.0 (147.4)*	382.0 (129.0)	477.0 (38.5)
HCO_3_ (mEq/L)	32.0 (1.9)*	31.6 (1.2)^#^	33.9 (1.5)

Data are reported as means (SD). PEEP 2, ventilated with a positive end-expiratory pressure (PEEP) of 2 cmH_2_O; PEEP 5, with a PEEP of 5 cmH_2_O, and PEEP 10, with a PEEP of 10 cmH_2_O. PCO_2_: partial pressure of carbon dioxide; PO_2_: partial pressure of oxygen. *P<0.05 *vs* PEEP 10; ^#^P<0.05 *vs* PEEP 10 (ANOVA).

No significant difference was found between the hemodynamic parameters of the different groups (Table 2), demonstrating that the groups presented the same hemodynamic stability. Higher saturation levels (hyperoxia) were also observed in animals with PEEP 5 and 10.

**Table 2:** Mean values of cardiopulmonary parameters and saturation for different final positive end-expiratory pressure (PEEP) groups.

	PEEP 2	PEEP 5	PEEP 10	P
Heart rate	464.0 (142.2)	382.0 (114.5)	366.0 (110.2)	0.11
Lactate (mg/dL)	0.6 (0.3)	0.6 (0.26)	0.6 (0.3)	0.99
Temperature (°C)	36.2 (0.8)	36.1 (0.7)	36.0 (0.8)	0.88
Perfusion (s)	0.2 (0.1)	0.2 (0.1)	0.08 (0.05)	0.11
SaO_2_ (%)	96.0 (7.4)	99.0 (8.2)	100.0 (0.09)	0.52

Data are reported as means (SD). PEEP 2, ventilated with a positive end-expiratory pressure (PEEP) of 2 cmH_2_O; PEEP 5, with a PEEP of 5 cmH_2_O, and PEEP 10, with a PEEP of 10 cmH_2_O. Data are reported as means (SD) (ANOVA). SaO_2_: peripheral oxygen saturation.

The need for pulmonary recruitment was evidently lower in the group that used PEEP 10 (14.3%). In the other groups, all animals (100%) required recruitment maneuvers due to the presence of atelectasis areas.

**Figure 1.**
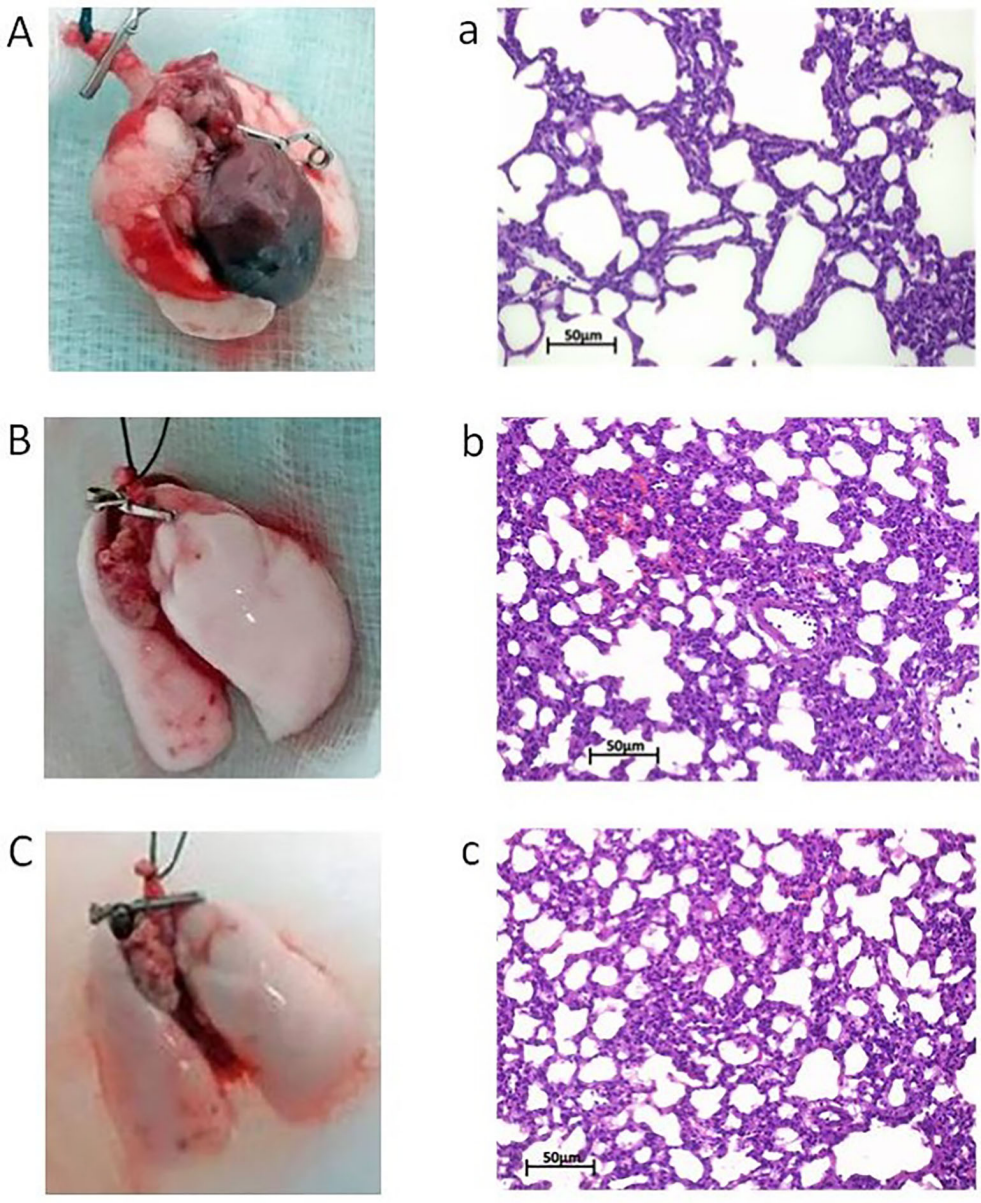
**A**/**a**: PEEP 2; **B**/**b**: PEEP 5; **C**/**c**: PEEP 10. Inflated lungs are smaller in **A** than in **B** and **C** and hemorrhagic areas are present only in **A**. Histomorphologically, alveolar sacs are more uniform and homogeneous in **b** and **c** compared to **a**. However, the focal septal thickening is more evident in **b**. PEEP: positive end-expiratory pressure at 2, 5, and 10 cmH_2_O. Scale bar: 50 μm.

The macroscopic and histological aspects of the lungs studied are illustrated in Figure 1. The inflated lungs were smaller in PEEP 2 than in PEEP 5 and PEEP 10, as well as hemorrhagic areas were present only in PEEP 2. Histomorphologically, the alveolar sack was more uniform and homogeneous in PEEP 5 and PEEP 10 compared to PEEP 2. However, focal septal thickening was more evident in PEEP 5.

**Figure 2.**
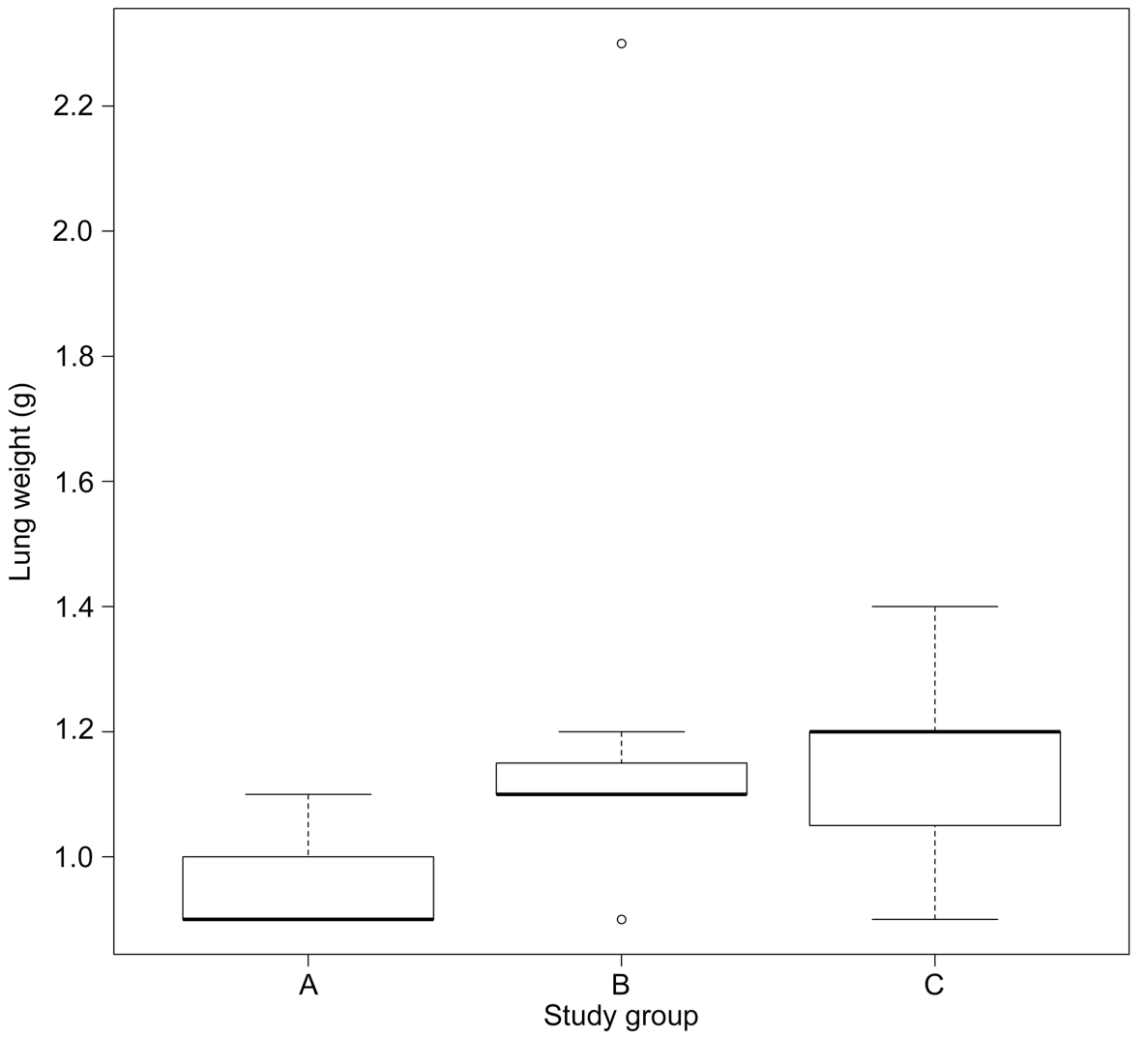
Box-plot of lung weight according to the study groups. Group A, ventilated with a positive end-expiratory pressure (PEEP) of 2 cmH_2_O; Group B, with a PEEP of 5 cmH_2_O, and Group C, with a PEEP of 10 cmH_2_O. Data are reported as medians and interquartile ranges.

The mean weights of the lungs were 0.96, 1.2, and 1.1 g, for PEEP 2, 5, and 10, respectively, with no significant difference between groups (P=0.11). The distribution of the lung weight data is presented in Figure 2.

## Discussion

The data demonstrated that protective ventilation is adequate for lung transplantation, although there are not many studies on different uses of PEEP during lung retrieval (15–18).

A multicenter randomized controlled trial of patients with beating hearts who were potential organ donors was performed at 12 European intensive care units. The study concluded that the use of a lung protective strategy (tidal volumes of 6–8 mL/kg, PEEP of 8–10 cmH_2_O) in potential organ donors increased the number of eligible lungs in the intensive care unit, but it did not evaluate the use of PEEP during lung retrieval (15).

The only study similar to the one presented here was conducted by Schumann et al. in 2010 (18). They studied the influence of different levels of PEEP (4 and 8 cmH_2_O) in an animal model (pig) during lung retrieval. However, they did not evaluate the need for lung recruitment and other factors involved, as different preservation perfusion rates hampered an adequate evaluation of the effects of PEEP (18).

Our study also evaluated the immediate effects of PEEP on the pulmonary structure during lung retrieval; however, the infusion rate of the preservation solution was a constant factor in our experiment, being the main object for studying the effect of PEEP.

Appropriate lung oxygenation of donor patients is an indicator of good prognosis, being required in most transplant programs. In 2015, only 20% of lungs suitable for donation were transplanted, and many of the unsuccessful cases were due to problems with ventilatory strategies, among them poor oxygenation (6,14).

Our model revealed that the use of PEEP 5 and 10 permitted achieving optimal PO_2_. There is a relative increase in the risk of death when the donor has PaO_2_/FiO_2_ <350 mmHg (13). In our study, PaO_2_/FiO_2_ <350 mmHg was observed with PEEP 2, but not with PEEP 5 and PEEP 10.

The animals with PEEP 10 presented hypercapnia and respiratory acidosis. However, these parameters were not evaluated during the decision of the viability of a lung for transplantation (19,20). Song et al. indicates that CO_2_ may play a positive role in a rat model of lung transplantation, with anti-inflammatory and anti-apoptotic properties and potent cytoprotection (21).

Regarding oxygen saturation, there was a clinical difference between animals in which PEEP 2 was used (96% saturation) and those receiving PEEP 5 (99% saturation, hyperoxia) and PEEP 10 (100% saturation, hyperoxia), with the same FiO_2_ (100%). Therefore, in PEEP 5 and 10 the oxygen supply could have been lower, a desirable fact since studies have demonstrated that a very high FiO_2_ may generate antioxidant substances that are toxic to the pulmonary parenchyma and may favor the onset of atelectasis (22–24).

The use of high PEEP is a well-established strategy in other ventilatory situations (25). Despite these data, the use of PEEP 2–5 is common in animal models (26) and in humans during lung retrieval (3,6). The reason why a high PEEP is not routinely used in lung transplantation has not been well described in the literature. The deleterious effects of PEEP reported in other types of patients as cardiovascular instability and excessive alveolar distention were not studied in lung donors (8).

In the present study, the animals remained hemodynamically stable, regardless of the PEEP administered, with all groups showing similar heart rate, perfusion, and lactate levels, data similar to those of the literature (4,7,13).

In addition, the present model showed that the use of PEEP 10 during lung retrieval caused lower occurrence of atelectasis and pulmonary recruitment was necessary in only one animal, whereas in the groups the received PEEP 2 and 5, all animals required the maneuver due to the presence of atelectasis.

The occurrence of atelectasis during lung retrieval and perfusion is very frequent and quite deleterious. This may lead to gasometrical values that do not reflect the real condition of the lung parenchyma, favoring very rapid perfusion with consequent pulmonary injury and edema and minimizing the effects of protective ventilation (5,6,25–27).

The use of pulmonary recruitment during retrieval is a common procedure. However, recruitment leads to the production of inflammatory mediators and pulmonary edema that may negatively affect the results of the transplant (28–35). In our study, PEEP 2 animals presented more lesions and PEEP 5 presented focal septal thickening, possibly characterizing greater pulmonary injury compared to animals treated with PEEP 10.

Some articles demonstrated that pulmonary edema, characterized by the variation of lung weight (14), could be reduced with the use of high PEEP (36–39). In our study, there was no statistical difference in lung weight. This finding is in disagreement with the literature, and we attributed the non-variation of the pulmonary weight to the fast lung removal, without storage, not allowing time for the manifestation of important pulmonary edema.

Our study confirmed the benefit of the use of high PEEP. Although other analyses of inflammatory substances and capillary permeability were not possible, this study demonstrated the differences between ventilatory strategies.

We suggest that the use PEEP 2 is not adequate during lung retrieval procedures. The use of PEEP 5 resulted in adequate oxygenation, but there were many atelectases requiring alveolar recruitment. Histological alterations associated with pulmonary injury were also observed. Animals with PEEP 10 presented good oxygenation without significant atelectasis and, therefore, did not require alveolar recruitment and presented histological characteristics that were more adequate than the other groups.

Therefore, the use of high PEEP (10 cmH_2_O) was feasible and possibly a beneficial strategy for the prevention of atelectasis and the optimization of oxygenation during lung retrieval. Clinical studies should be performed to confirm this hypothesis.
